# Immersive Virtual Reality Exercise: Effects on Cortisol, Quality of Life, Cognitive Function, and Psychological Symptoms in Fibromyalgia

**DOI:** 10.3390/medicina62030446

**Published:** 2026-02-27

**Authors:** Gonzalo Arias-Álvarez, María Santamera-Lastras, Dina Guzmán-Oyarzo, Waldo Osorio-Torres, Benjamín Parada-Norambuena, Daniel Pecos-Martín, Jesús G. Ponce González, José Gómez-Pulido, Claudio Carvajal-Parodi

**Affiliations:** 1 Departamento de Medicina y Especialidades Médicas, Universidad de Alcalá, 28805 Alcalá de Henares, Spain; maria.santamera@uah.es; 2 Escuela de Kinesiología, Facultad de Ciencias de la Rehabilitación y Calidad de Vida, Universidad San Sebastián, Concepción 4080871, Chile; wosoriot@docente.uss.cl (W.O.-T.); bparadan@docente.uss.cl (B.P.-N.); claudio.carvajal@uss.cl (C.C.-P.); 3 Escuela de Tecnología Médica, Facultad de Ciencias, Universidad San Sebastián, Lientur 1439, Concepción 4030000, Chile; dina.guzman@uss.cl; 4 Departamento de Enfermería y Fisioterapia, Universidad de Alcalá, 28801 Alcalá de Henares, Spain; daniel.pecos@uah.es; 5 ExPhy Research Group, Department of Physical Education, Instituto de Investigación e Innovación Biomédica de Cádiz (INiBICA), Universidad de Cádiz, 11002 Cádiz, Spain; jesusgustavo.ponce@uca.es; 6 Departamento de Ciencias de la Computación, Universidad de Alcalá, 28805 Alcalá de Henares, Spain; jose.gomez@uah.es; 7 Escuela de Doctorado de la Universidad de Cádiz, Facultad de Ciencias de la Educación, Universidad de Cádiz, 11510 Puerto Real, Spain

**Keywords:** fibromyalgia, immersive virtual reality, therapeutic exercise

## Abstract

*Background and Objectives*: Fibromyalgia (FM) is a chronic and complex condition characterized by widespread pain, fatigue, psychological burden, and cognitive impairment, posing significant challenges for treatment. Immersive virtual reality exercise (iVRE) has been proposed as an innovative therapeutic approach to increase adherence, motivation, and multidimensional benefits, but evidence in FM remains limited. This study aimed to evaluate the effects of a six-week iVRE program on cortisol levels, quality of life, cognitive function, and psychological symptoms in women with FM. *Materials and Methods*: A quasi-experimental pre–post design was conducted with 21 women (mean age 48.1 ± 10.7 years) diagnosed with FM, who completed twelve 30 min sessions of iVRE using Oculus Quest 2™ and the FitXR platform. Outcomes assessed pre- and post-intervention included salivary cortisol (ELISA), quality of life (FIQR), emotional status (DASS-21), and cognitive function (MoCA). Adherence and safety were monitored throughout. *Results*: The intervention was well tolerated, with no adverse events and 100% adherence. Statistically significant improvements were observed in FIQR scores (*p* < 0.001, d = 3.54), depression (*p* < 0.001, d = 1.19), anxiety (*p* < 0.001, d = 1.39), and stress (*p* < 0.001, d = 2.28). Cognitive performance improved significantly, with higher MoCA total scores (*p* < 0.001, d = 1.52) and better outcomes in visuospatial ability, language, and delayed recall domains. No significant changes were detected in salivary cortisol levels (*p* = 1.000). *Conclusions*: A six-week iVRE program is safe and feasible, promoting clinically relevant improvements in quality of life, emotional well-being, and cognitive function in women with FM, despite the absence of changes in cortisol. These findings highlight iVRE as a promising complementary therapeutic strategy within multidisciplinary FM management, warranting further controlled studies with larger samples and long-term follow-up to confirm its efficacy and explore underlying mechanisms.

## 1. Introduction

Fibromyalgia (FM) is a complex and debilitating rheumatic disorder that affects multiple body systems [1]. This condition has a prevalence ranging from 2% to 4% [2], primarily affecting women between 20 and 50 years of age [3]. Its main symptoms include widespread musculoskeletal pain, persistent fatigue, sleep disturbances, and a high emotional burden, often involving elevated levels of anxiety and depression [4,5,6]. In addition to these manifestations, patients may also experience “fibro fog” [7], characterized by difficulties in concentration, short-term memory loss, and challenges with multitasking [8]. These symptoms have a significant impact on quality of life and overall well-being, posing a major therapeutic challenge in clinical practice for healthcare professionals involved in their treatment [9].

Several authors have reported that the pathophysiology of FM involves dysfunction in central pain modulation mechanisms [10], as well as dysregulation of the hypothalamic–pituitary–adrenal (HPA) axis [11], which regulates the stress response [12]. This condition is often reflected in altered cortisol levels, which may contribute both to increased pain perception and to worsening emotional and cognitive states [13].

In light of this, it is essential to develop therapeutic strategies that address the multiple dimensions of FM, including pain reduction, functional improvement, and psychological well-being [14]. However, participation and adherence to conventional programs are often hindered by the fluctuating symptomatology of the disease and by emotional barriers that many individuals with FM face when engaging in regular physical activity [15,16,17].

In this context, immersive virtual reality (IVR) has emerged as an innovative therapeutic alternative to enhance the experience of therapeutic exercise [18,19]. By generating engaging and adaptable virtual scenarios, IVR promotes active participation in exercise programs, encouraging greater adherence and enjoyment of physical activity [20]. Moreover, the multisensory immersive nature of this technology may exert a modulatory effect on psycho-emotional and cognitive variables in individuals with FM [21,22,23].

Although interest in the use of IVR in therapeutic contexts has been increasing, the available evidence regarding the effects of IVR-based therapeutic exercise in patients with FM remains limited [23]. Therefore, it is necessary to further explore its impact not only on traditional clinical outcomes, such as quality of life and psychological symptoms, but also on physiological stress-related parameters, such as cortisol levels. The present study aimed to evaluate the effects of a therapeutic exercise program complemented with IVR on cortisol levels, quality of life, cognitive function, and symptoms of stress, anxiety, and depression in women diagnosed with FM, thereby providing new insights into the potential of this technology as a therapeutic tool.

## 2. Materials and Methods

### 2.1. Design and Subjects

A quasi-experimental study with a pre–post design and no control group was conducted. The intervention took place at Universidad San Sebastián, Bío-Bío Region, Chile. The study was registered at www.clinicaltrials.gov (Identifier: NCT06658223) and approved by the Scientific Ethics Committee of Universidad San Sebastián (Approval No. 73-24). The research was conducted in accordance with the ethical principles outlined in the 1964 Declaration of Helsinki and its later amendments [24]. The sample was selected through convenience sampling, applying the following criteria:

(i) Inclusion: Individuals over 18 years of age diagnosed with FM, with the diagnosis previously established by a medical professional according to the updated diagnostic criteria for FM [25].
(ii) Exclusion: Pregnant or breastfeeding women, as well as individuals with cancer-related pain, uncontrolled metabolic disorders, vertigo, or other conditions that could interfere with the intervention. Participants with physical or cognitive limitations that hindered effective communication with the research team or prevented the proper execution of the sessions were also excluded.

To determine the minimum required sample size, G*Power software (version 3.1.9.7) was used, considering a paired-sample *t*-test, a significance level of 5%, a statistical power of 80%, and an estimated effect size of 0.65, based on previous studies with similar interventions. Under these parameters, a minimum of 19 participants was required. However, the sample was expanded to 21 participants to account for potential dropouts or incomplete records, thereby ensuring greater robustness in the analysis of the results.

Patients who met the inclusion criteria were invited to participate at the Physiotherapy Center of Universidad San Sebastián. Once the exclusion criteria were verified and informed consent was obtained, the researchers administered a structured clinical form to collect sociodemographic data, health history, and information on pharmacological treatment.

### 2.2. Intervention (iVRE)

The intervention consisted of a therapeutic exercise program using IVR over six weeks, with two sessions per week, totaling twelve sessions per participant. Each session lasted approximately 30 min, comprising a 10 min warm-up phase followed by 20 min of exercise using the FitXR game (developed by FITAR LIMITED, London, UK) through the Oculus Quest 2™ device (Meta Platforms, Inc., Menlo Park, CA, USA). This dosage was selected based on clinical recommendations for individuals with chronic pain, prioritizing safety and tolerance to physical effort. Several authors have suggested session durations between 10 and 30 min to minimize adverse effects and enhance adherence to treatment [26,27,28].

The warm-up phase consisted of 10 min of aerobic exercise at a self-selected intensity on a cycle ergometer, aimed at preparing participants for higher-intensity activity. After the warm-up, participants proceeded with the IVR-based therapeutic exercise. During these sessions, they were able to choose from different virtual environments as well as the type of game to be performed. They then selected a difficulty level, with three options: beginner, intermediate, or advanced. The distinctions between these levels were defined by the complexity of movements, the intensity of physical effort required, and the speed of execution, thereby allowing for gradual progression according to individual tolerance.

Each virtual reality session was divided into three phases:

Phase 1: During the first five minutes, general mobility exercises were performed targeting the upper and lower limbs as well as the spine. The aim of this stage was to prepare the body for the main activity, promoting progressive joint and muscle activation. Guidance was provided by a virtual avatar, which instructed participants through clear verbal cues and real-time visual demonstrations, thereby ensuring safe and technically correct execution of each movement. This interactive interface helped maintain attention, facilitated understanding of motor sequences, and promoted autonomy throughout the session.
Phase 2: The second phase corresponded to the main activity within the virtual environment. In this stage, participants performed the exercise standing and could choose among three available modalities: aerobox, dance fitness, or zumba. Each option offered a distinct focus in terms of rhythm, coordination, and physical effort, allowing the experience to be adapted to individual interests and capabilities while maintaining a common therapeutic goal. During the activity, participants were guided by a virtual trainer who appeared through video and voice instructions. This trainer provided clear, sequential guidance combined with motivational prompts aimed at sustaining engagement and concentration throughout the session. The digital environment also delivered immediate feedback through auditory and visual stimuli, including floating targets indicating the required movements. These elements facilitated motor synchronization, sequence tracking, and real-time performance perception, reinforcing both the playful and therapeutic components of the experience.
Phase 3: The final phase of each session was devoted to a cool-down period lasting approximately five minutes. During this stage, participants performed gentle joint mobility exercises focused on the spine and extremities, aimed at promoting progressive recovery after the main activity. This phase was complemented with a series of muscle stretches designed to reduce accumulated tension and prevent post-exercise discomfort. In addition, conscious breathing exercises were incorporated to foster participant relaxation.

During the gaming experience, participants received immediate feedback through a real-time scoring system, calculated based on the accuracy, intensity, and consistency of their movements. This system not only enhanced motivation during the activity but also enabled continuous performance assessment. At the end of each session, a performance category was assigned, ranging from Beginner to Legendary, according to the total score achieved. In addition, a summary was displayed with key indicators such as total points accumulated, activity time, an estimate of calories burned, and a comparison with the performance of other users.

### 2.3. Assessment of Outcomes

The assessments were conducted at the Physiotherapy Center of Universidad San Sebastián. Sociodemographic and clinical variables were identified through a questionnaire administered in a personal interview, with the information recorded in a clinical file. The questionnaires were administered by physiotherapists specialized in musculoskeletal rehabilitation, each with over 15 years of clinical experience. All tests were performed both before and after the sixth week of treatment.

#### 2.3.1. Impact of FM on Quality of Life

The impact of FM on quality of life was assessed using the FIQR questionnaire. This self-administered instrument measures the difficulty in performing activities of daily living, the overall impact of the disease, and the intensity of symptoms experienced during the previous week. The total score ranges from 0 to 100, with higher values indicating greater symptom severity and a higher level of functional disability [29,30].

#### 2.3.2. Stress, Anxiety and Depression

Levels of stress, anxiety, and depression were assessed using the DASS-21 questionnaire. This self-administered, dimensional instrument specifically examines three emotional domains: stress, anxiety, and depression. It consists of 21 items equally distributed across the three subscales, each with four response options scored from 0 to 3, according to the frequency or intensity of the symptom experienced. For each subscale, the score is calculated by summing the corresponding responses and multiplying the total by two, which allows the classification of symptomatology into five categories: normal, mild, moderate, severe, or extremely severe. Its use is widely supported by evidence confirming its reliability and validity for both the detection and monitoring of emotional symptoms in clinical and research settings [31,32].

#### 2.3.3. Cortisol

Salivary cortisol levels were measured using the commercial Cortisol Saliva ELISA kit (REF RE52611, IBL International GmbH^®^, Hamburg, Germany), based on a solid-phase competitive immunoassay [33,34]. Prior to sample collection, participants were instructed to avoid food, beverages, chewing gum, or tooth brushing for at least 60 min, following the manufacturer’s recommendations. Samples were obtained by direct expectoration into sterile tubes, collecting at least 1 mL of additive-free saliva while avoiding visible blood contamination. The samples were then frozen at −20 °C until analysis. Before the assay, samples were thawed, homogenized, and centrifuged at 3000× *g* for 10 min to remove particles. The ELISA was performed according to the manufacturer’s instructions. Briefly, 50 µL of each sample, standard, and control were pipetted into antibody-coated wells, followed by the addition of 100 µL of enzyme conjugate. The plate was incubated for 2 h at room temperature with orbital shaking. After washing with diluted wash buffer, 100 µL of TMB substrate was added and incubated for 30 min. The reaction was then stopped with 100 µL of stop solution, and absorbance was measured at 450 nm. Cortisol concentrations were determined by interpolation from a standard curve generated with known concentrations (0–3 µg/dL). A four-parameter logistic (4PL) regression was applied to fit the curve. All procedures were carried out in the Research Laboratory of Universidad San Sebastián.

#### 2.3.4. Adverse Events

The occurrence of adverse events associated with the use of iVRE was monitored by a physiotherapist responsible for supervising the implementation of the intervention protocol. Specific symptoms evaluated included dizziness, headaches, neck pain, and general discomfort [35]. These data were recorded on a monitoring form, with each symptom documented as either “present” or “absent.”

### 2.4. Statistical Analysis

Participants who completed all intervention protocols were included in the data analysis. Statistical analyses were performed using JASP^®^ software (version 0.18.3). The Shapiro–Wilk test was applied to assess the normality of quantitative variables. For data with a normal distribution (*p* > 0.05), paired Student’s t-tests were used. For variables that did not meet the normality assumption (*p* ≤ 0.05), the Wilcoxon signed-rank test was applied. In addition, effect sizes were calculated using Cohen’s d for *t*-tests and the rank-biserial correlation for Wilcoxon tests. A significance level of *p* < 0.05 was considered for all analyses. Given the exploratory nature of this pilot study and the small sample size, no correction for multiple comparisons was applied. Future studies with larger samples should include confidence intervals and appropriate correction methods to reduce the likelihood of Type I errors.

## 3. Results

**Figure 1:**
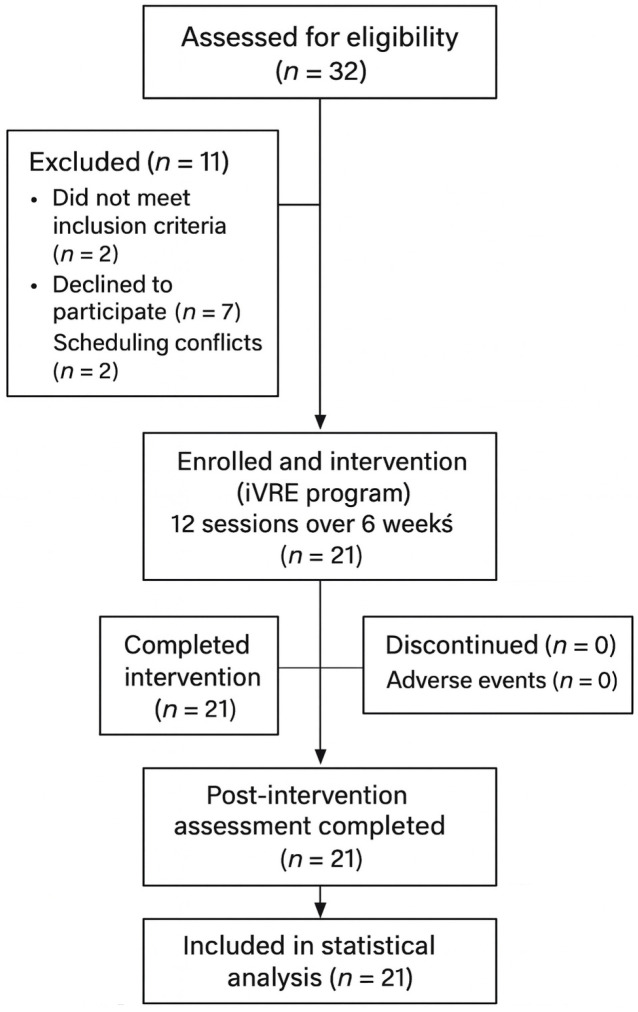
Flow diagram of participants through the study (TREND). Assessed for eligibility (*n* = 32); excluded (*n* = 11: did not meet inclusion criteria (*n* = 2), declined to participate (*n* = 7), scheduling conflicts (*n* = 2)); enrolled and received intervention (iVRE program; 12 sessions over 6 weeks) (*n* = 21); completed intervention (*n* = 21); discontinued (*n* = 0); adverse events (*n* = 0); post-intervention assessment completed (*n* = 21); included in statistical analysis (*n* = 21).

A total of 32 individuals diagnosed with FM were assessed, of whom 30 met the inclusion criteria. Nine participants were excluded for personal reasons unrelated to the intervention protocol, including scheduling conflicts (*n* = 2), acute illnesses not related to FM (*n* = 5), and scheduling delays that could not be accommodated (*n* = 2). Ultimately, 21 participants (100% women) completed the intervention protocols, yielding a 70% retention rate and 100% adherence (Figure 1). The mean age was 48.1 ± 10.7 years. According to the World Health Organization guidelines, 76.2% of the sample was classified as sedentary, while 19% engaged in low-intensity physical activity [36]. The most commonly used medications were NSAIDs (100%), antidepressants (71.4%), and GABAergic drugs (71.4%). Table 1 shows the sociodemographic characteristics of the participants.

The intervention was carried out successfully, with no adverse events reported during the study. After verifying the assumption of normality with the Shapiro–Wilk test, it was determined that the initial and final FIQ-R (*p* = 0.118 and *p* = 0.518), DASS-21 Depression (*p* = 0.392 and *p* = 0.267), DASS-21 Stress (*p* = 0.009 and *p* = 0.060), DASS-21 Anxiety (*p* = 0.271 and *p* = 0.181), and overall MoCA pre and post (*p* = 0.562 and *p* = 0.253) presented distributions compatible with normality; therefore, paired Student’s t-tests were applied. In all cases, statistically significant differences were found: reductions in FIQ-R scores (*p* < 0.001, d = 3.680), depression levels (*p* < 0.001, d = 1.196), stress (*p* < 0.001, d = 2.287), and anxiety (*p* < 0.001, d = 1.392), as well as an increase in the total MoCA score (*p* < 0.001, d = −1.524), indicating an overall improvement in the evaluated cognitive functions. These results are presented in Table 2.

For variables that did not show a normal distribution according to the Shapiro–Wilk test, the Wilcoxon signed-rank test was applied. No significant differences were found in pre- and post-intervention cortisol levels (*p* = 1.000, r = −0.011), in the identification subscale (*p* = 1.000, r = 1.000), attention (*p* = 0.146, r = −0.451), or orientation (*p* = 0.072, r = −1.000). In contrast, statistically significant improvements were observed in the visuospatial subscale of the MoCA (*p* = 0.009, r = −0.800), in the language domain (*p* = 0.003, r = −1.000), and in delayed recall (*p* < 0.001, r = −0.865), suggesting a positive impact of the intervention on specific domains of cognitive function. These results are presented in Table 3.

## 4. Discussion

This study aimed to evaluate the effects of an immersive virtual reality (IVR) exercise program on cortisol levels, quality of life, cognitive function, and symptoms of stress, anxiety, and depression in women diagnosed with FM.

The findings provide preliminary yet promising evidence regarding the potential benefits of a six-week IVR-supported therapeutic exercise program in this population. Participation in the intervention was associated with improvements in quality of life, emotional well-being, and specific cognitive domains. These results suggest that iVRE may represent a feasible and engaging complementary approach within the multidisciplinary management of fibromyalgia, although causal interpretations should be avoided due to the single-group pre–post design.

In particular, FIQR scores reflected a significant reduction in the overall impact of FM after completion of the program. These results align with previous research highlighting the potential of virtual exercise to modulate pain perception and support daily functioning in individuals with FM [37,38,39].

In contrast to more conventional interventions, immersive virtual reality integrates playful, personalized, and motivational elements that enhance the therapeutic experience [40,41], potentially strengthening treatment adherence by reducing psychological barriers commonly associated with maintaining regular physical activity [42].

In the emotional domain, clinically relevant reductions were observed in stress, anxiety, and depression levels, as assessed by the DASS-21. These changes may be attributed, at least in part, to the distractive effect and the immersive experience provided by virtual reality, which are capable of positively modulating the limbic system and the emotional responses associated with pain [43,44,45]. This proposition is consistent with the literature describing the ability of virtual reality to modulate neural circuits involved in the perception and regulation of emotions [46,47,48].

With regard to cognitive function, the data revealed a significant increase in the overall MoCA score, with notable improvements in domains such as visuospatial ability, language, and delayed recall. In this context, several authors have suggested that virtual reality–based technologies may enhance neuroplasticity and promote cognitive improvement by inducing neural changes and adaptations, as well as by increasing synaptic density, thereby facilitating the acquisition of new skills [46,49,50]. However, these findings should be interpreted with caution, given the heterogeneity of methodological designs, the limited replication and extrapolation of results across studies, and, above all, the multifactorial nature underlying the occurrence of cognitive changes [51,52,53]. Nevertheless, these findings gain relevance considering the high prevalence of cognitive impairments among individuals with fibromyalgia [6,54,55]. Although the pathophysiology of these deficits has not been fully elucidated [56], neuroendocrine alterations involving the hypothalamic–pituitary–adrenal (HPA) axis have been proposed. However, in the present study no significant changes were observed in salivary cortisol levels, suggesting that the emotional and cognitive benefits may not rely, at least in the short term, on the normalization of the endocrine stress response.

The absence of changes in cortisol does not diminish the clinical effects observed. Rather, it highlights the need for longitudinal studies of greater duration and intensity to examine whether sustained immersive exercise may modulate the HPA axis or other stress-related markers. In this regard, incorporating complementary measures, such as heart rate variability or levels of proinflammatory cytokines, would contribute to a more comprehensive understanding of the underlying physiological mechanisms.

It is also noteworthy that no adverse events were reported during the intervention, supporting the safety and tolerability of this type of technology in clinical settings. The ability to adapt the virtual environment, difficulty level, and exercise characteristics enabled an individualized approach that respected each participant’s capacities—an essential aspect of managing conditions as complex as FM.

Although the observed effect sizes were large, these values should be interpreted with caution. The combination of a small sample size, single-group pre–post design, and within-subject analysis may inflate the magnitude of statistical effects. This overestimation is commonly reported in pilot studies, particularly when no control group is included. Therefore, larger randomized controlled trials are required to confirm these preliminary findings and to determine the reproducibility and long-term sustainability of the observed effects.

Despite these contributions, this study presents several important limitations. First, given its single-group pre–post design, it is not possible to rule out that part of the improvements observed may be influenced by nonspecific factors such as placebo effects, positive expectations, or natural symptom variation. Therefore, the results should be interpreted with caution and considered preliminary evidence that needs to be confirmed through randomized controlled trials designed to isolate these potential sources of bias. The absence of long-term follow-up limits the ability to determine the sustainability of the effects. In addition, concomitant treatments, such as pharmacological use, were not controlled for, which may have influenced the results. Cortisol assessment, conducted at only two time points and without adjustment for circadian rhythm, may not accurately reflect modifications in the physiological stress response. The relatively small sample size further restricts the generalizability of the findings. Nevertheless, the consistency of the observed changes across different domains, together with the pre–post design, supports the plausibility of the reported benefits. Future research employing controlled designs, larger samples, and extended follow-up will be essential to validate and expand upon these findings.

## 5. Conclusions

The findings of this study suggest that a therapeutic exercise program based on immersive virtual reality may yield significant benefits in quality of life, emotional status, and specific domains of cognitive function in women diagnosed with fibromyalgia. The intervention was well tolerated, with no adverse events reported, supporting its safety and feasibility in clinical settings. Although no changes were observed in salivary cortisol levels, the results support the consideration of immersive virtual reality exercise as a promising complementary tool within the multidisciplinary management of fibromyalgia. However, it should be noted that the observed benefits may arise from mechanisms shared with other interventions, such as mind–body exercise programs or psychological therapies based on mindfulness and acceptance, which also promote engagement, positive affect, and cognitive reframing. Therefore, these findings should be interpreted with caution and within the broader context of multimodal care. Further longitudinal and comparative studies are required to determine the persistence of these effects and to clarify whether iVRE confers additional or distinct advantages beyond existing interventions.

## Figures and Tables

**Table 1:** Sociodemographic characteristics of the sample.

Age (Years)	Mean	SD
	48.1	10.7
Sex	*n*	%
Women	21	100
Men	0	0
Employment status	*n*	%
Employed	7	33.3
Freelance	3	14.4
Homeowner	10	47.6
Unemployed	1	4.7
Physical activity	*n*	%
Sedentary	16	76.2
Low intensity	4	19.0
Moderate intensity	1	4.8
High intensity	0	0
Drugs	*n*	%
NSAIDs	21	100
Opioids	10	47.5
GABAergics	15	71.4
Antidepressants	15	71.4

SD: standard deviation, *n*: sample, %: percentage, NSAIDs: nonsteroidal anti-inflammatory drugs; GABA: gamma-aminobutyric acid. Units are expressed where applicable.

**Table 2:** Effects of the iVRE program on FIQR, DASS-21 and MoCA.

Outcome	Pre-Test Media	SD	Post-Test ^1^ Media	SD	*p*-Value	Cohen’s *d*
FIQR	70.48	22.95	41.66	19.49	**<** **0** **.001**	3.54
DASS-21 depression	11.23	5.58	6.90	2.96	**<** **0** **.001**	1.19
DASS-21 anxiety	12.19	5.85	7.19	3.69	**<** **0** **.001**	1.39
DASS-21 stress	14.23	5.53	7.42	3.26	**<** **0** **.001**	2.28
MoCA	21.85	3.77	26.04	2.08	**<0.001**	1.52

^1^ Measurement after six weeks of iVRE. SD: standard deviation, MoCA: Montreal Cognitive Assessment, FIQR: Revised Fibromyalgia Impact Questionnaire, DASS-21: Depression Anxiety Stress Scales–21 items, iVRE: Immersive virtual reality exercise.

**Table 3:** Effects of the iVRE program on salivary cortisol levels and MoCA Subscales.

Outcome	Pre-Test Media	SD	Post-Test ^1^ Media	SD	W	Z	*p*-Value	r
Cortisol	0.22	0.33	0.14	0.07	**45.0**	−0.03	1.00	−0.01
MoCA Subscales								
Visuospatial	2.76	1.75	3.95	0.86	**10.5**	−2.63	**<0.00**	−0.80
Naming	2.95	0.21	2.90	0.30	**1.0**	1.00	1.00	1.00
Attention	4.47	1.50	4.85	0.85	**25.0**	−1.43	0.14	−0.45
Language	2.14	0.79	3.00	0.54	**0**	−2.93	**<0.00**	−1.00
Delayed recall	1.95	1.11	3.38	1.11	**11.5**	−3.22	**<0.01**	−0.86
Orientation	5.76	0.43	5.95	0.21	**0**	−1.82	0.07	−1.00

^1^ Measurement after six weeks of iVRE. MoCA: Montreal Cognitive Assessment, SD: standard deviation, W: Wilcoxon Signed-Rank Statistic, Z: Standardized Test Statistic (Z-score), r: Effect Size (Rank-Biserial Correlation), iVRE: Immersive virtual reality exercise.

## Data Availability

The data presented in this study are available on request from the corresponding author. The data are not publicly available due to privacy and ethical restrictions related to participant confidentiality.

## Abbreviations

The following abbreviations are used in this manuscript:

FM	Fibromyalgia
iVRE	Immersive virtual reality exercise
IVR	Immersive virtual reality
HPA	Hypothalamic–pituitary–adrenal (axis)
FIQ-R	Revised Fibromyalgia Impact Questionnaire
DASS-21	Depression Anxiety Stress Scales–21 items
MoCA	Montreal Cognitive Assessment
SD	Standard deviation
n	Sample
%	Percentage
NSAIDs	Nonsteroidal Anti-Inflammatory Drugs
4PL	Four-parameter logistic
TMB	Tetramethylbenzidine
VCM	Vice-Rector’s Office for University Outreach
